# HGGA: hierarchical guided genome assembler

**DOI:** 10.1186/s12859-022-04701-2

**Published:** 2022-05-07

**Authors:** Riku Walve, Leena Salmela

**Affiliations:** Department of Computer Science, Helsinki Institute for Information Technology HIIT, University of Helsinki, Helsinki, Finland

**Keywords:** Genome assembly, Genetic linkage maps

## Abstract

**Background:**

*De novo* genome assembly typically produces a set of contigs instead of the complete genome. Thus additional data such as genetic linkage maps, optical maps, or Hi-C data is needed to resolve the complete structure of the genome. Most of the previous work uses the additional data to order and orient contigs.

**Results:**

Here we introduce a framework to guide genome assembly with additional data. Our approach is based on clustering the reads, such that each read in each cluster originates from nearby positions in the genome according to the additional data. These sets are then assembled independently and the resulting contigs are further assembled in a hierarchical manner. We implemented our approach for genetic linkage maps in a tool called HGGA.

**Conclusions:**

Our experiments on simulated and real Pacific Biosciences long reads and genetic linkage maps show that HGGA produces a more contiguous assembly with less contigs and from 1.2 to 9.8 times higher NGA50 or N50 than a plain assembly of the reads and 1.03 to 6.5 times higher NGA50 or N50 than a previous approach integrating genetic linkage maps with contig assembly. Furthermore, also the correctness of the assembly remains similar or improves as compared to an assembly using only the read data.

## Background

*De novo* genome assembly asks to reconstruct the genomic sequence of a new previously unsequenced organism given a set of sequencing reads. The advances in long read sequencing technologies have allowed for complete or near complete assemblies of bacterial and small genomes [1–3] but assembling larger genomes such as vertebrates and plants typically yields a set of contigs instead of the complete genome of the organism. Thus resolving the large scale structure of these genomes requires additional long range data such as genetic linkage maps, optical maps, or Hi-C data [4].

Typically additional long range data is used to order the contigs into scaffolds. A genetic linkage map consists of a set of markers such as single nucleotide variations (SNVs). The markers are divided into chromosomes and their partial order within a chromosome is known. Chromonomer [5] uses a genetic linkage map to scaffold contigs and it also detects misassemblies and can correct these based on a genetic linkage map. Similarly, Lep-Anchor [6] can detect and correct chimeric contigs based on a genetic linkage map and it can anchor contigs into chromosomes using a genetic linkage map and additional information such as contig-contig and read alignments.

Optical maps are another type of physical maps that can be used to scaffold contigs. Optical maps are produced by elongating DNA molecules on a plate, applying a restriction enzyme that cuts or nicks the DNA molecules at specific restriction sites, and then imaging the cut or nicked molecules. SewingMachine [7] uses a single optical map to scaffold contigs, whereas OMGS [8] can simultaneously use several optical maps produced with different enzymes.

Some methods integrate the long range data directly into contig assembly. AGORA [9] uses optical maps to guide the contig building from a de Bruijn graph. KOOTA [10] maps reads to an optical map and uses the mapping positions to produce a positional de Bruijn graph which is less tangled than a regular de Bruijn graph. Kermit [11] maps reads to a genetic linkage map and then uses this information to remove edges that conflict with a genetic linkage map from the assembly graph of miniasm [1]. OpticalKermit [12] is a modification of Kermit to use optical maps instead of genetic linkage maps.

All previous methods that integrate the long range data directly to contig assembly build the assembly graph for the whole set of reads and use the long range data to disentangle the graph. Here we propose a different approach. We first map the individual reads to the long range data and then cluster the reads based on these mappings. Each cluster is then assembled independently and finally we join the clusters in a hierarchical manner to produce a complete assembly. We implement our approach for genetic linkage maps in a tool called HGGA (Hierarchical Guided Genome Assembler) and show that on real and simulated Pacific Biosciences reads and genetic linkage maps, HGGA produces a more contiguous assembly with less contigs and from 1.2 to 9.8 times higher NGA50 or N50 than a plain assembly of the reads and 1.03 to 6.5 times higher NGA50 or N50 than a previous approach integrating genetic linkage maps with contig assembly. Our approach is also easy to parallelize as the long range data naturally divides the reads into clusters which can be assembled independently in a parallel fashion.

### Related work

The *de novo* assembly problem asks to reconstruct a genome from a set of sequencing reads. The two most popular approaches to solve it are the overlap-layout-consensus approach and the de Bruijn graph based approach. In the overlap-layout-consensus approach first overlaps between reads are found. These can be represented in the form of an overlap graph where the nodes are the reads and there is an edge between two reads if they overlap. The overlap graph can then be simplified by removing transitive edges. The resulting graph is called the string graph [13]. In the layout phase, contigs are formed as paths in the string graph. Finally the consensus phase determines the base sequence of the contigs based on the reads. The alternative approach based on de Bruijn graphs first extracts all *k*-mers, i.e. *k* bases long sequences, that occur in the reads. These *k*-mers then form the nodes of the de Bruijn graph and there is an edge between two nodes if the *k*-mers overlap by $k-1$ bases. Contigs are typically reported as non-branching paths in the de Bruijn graph. The term assembly graph is often used to refer to both string graphs and de Bruijn graphs.

Reference guided assembly gives an attractive alternative to *de novo* assemblies. Here we are also given a reference sequence, against which we can compare our input reads. Schneeberger et al. [14] proposed a reference guided assembly approach which was further developed by Lischer and Shimizu [15]. They first map the reads against the reference. Based on the mappings the reads are divided into overlapping superblocks which are assembled independently into contigs and unmapped reads are also assembled separately into contigs. All these contigs are then joined into a set of supercontigs. AlignGraph [16] implements an alternative approach. First, all reads are assembled into contigs. Then the contigs and paired end reads are aligned against the reference sequence. Based on these alignments the contigs are further extended and joined into longer contigs.

In our previous work, we introduced Kermit [11], a method for guiding an assembly with a genetic linkage map instead of a reference sequence. Genetic linkage maps are a technique to orient and place contigs within a chromosome and to detect misassembled contigs. The genetic linkage maps themselves consist of genetic markers. The markers are divided into chromosomes and within each chromosome, the markers are further placed into bins. The order of the bins within a chromosome is known but the order of markers within a bin is not known.

The markers in the map are derived from a set of variations, such as single-nucleotide variations. The variations are found from a sequenced cross, a population of related individuals. Variations that are close to each other in the genome are likely to be inherited together. Genetic linkage maps can therefore be constructed by genotyping the individuals in the cross and examining the probabilities of variations being inherited together. Kermit colors the read set by mapping them to a genetic linkage map and then removes edges from the assembly graph that are not consistent with the coloring. While the same method theoretically extends to any guide data that can be represented as a linear ordering for reads, such as optical maps [12], it fails to generalize to non-linear guide data.

Kermit uses miniasm [1] for both assembly graph construction and genome assembly using the graph. We will be similarly using miniasm heavily here for easy comparison. Miniasm first uses minimap to find overlaps between the reads. Based on the overlaps it then creates a string graph by removing transitive edges. The graph is then cleaned by removing tips and popping bubbles. Finally miniasm reports unitigs, i.e. non-branching paths, in the resulting graph as contigs. Miniasm does not implement a consensus phase and thus the error rate of contigs produced by miniasm is the same as the error rate of the reads.

## Results

### Overview of our method

The input to our method is a set of reads and guide data describing the overall structure of the genome. First, we use the guide data to cluster the reads into multiple hierarchical trees where the set of reads is split into the leaves. Each leaf thus consists of a set of reads originating from nearby locations of the genome according to the guide data and the leaves are joined into multiple hierarchical binary trees according to the clustering. As the genomic distance between reads in different chromosomes is not defined, multiple trees, one for each chromosome, need to be used to cover a full multi-chromosomal genome.

We have implemented our method using genetic linkage maps as guide data. A genetic linkage map is usually constructed with respect to a draft assembly. Thus in this case the input consists of a set of reads, the genetic linkage map, and the draft assembly that has been used to construct the genetic linkage map. The draft assembly is used only for calling SNVs which become the markers of the genetic linkage map. The markers are ordered based on the observed patterns of inheritance and thus the ordering is done independently of the draft assembly.

The reads are localized on the genetic linkage map by aligning them to the draft genome and checking which markers of the genetic linkage map are closest to the alignment. The read is classified using the set of markers that are roughly equidistant from the closest marker. The classifications define a partial order for the reads and can thus be directly used to split the reads into leaves and to construct the hierarchical tree.

**Fig. 1:**
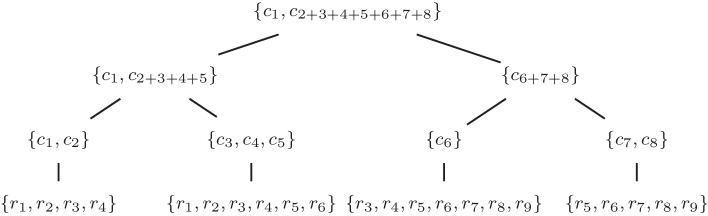
An example of how different points of the hierarchy affect the assembly. At the bottom raw reads are assembled into contigs in the leaf nodes. Each internal node then takes the contigs and further merges the contigs from its children together

Next, we assemble the reads using the hierarchical tree. The assembly pipelines for leaf nodes and internal nodes are different. For leaf nodes, the input is raw long reads and as such, we use existing tools for assembling and error correcting the reads into polished contigs for further assembly in the hierarchy. Finally, for each internal node, we take the contigs from the child nodes and assemble them. Internal nodes take the error corrected contigs as input and output longer super-contigs, so we use a simple greedy assembly algorithm to combine the input contigs. The final assembly is produced at the root of the tree. The assembly process is shown in Fig. 1.

### Data sets and evaluation

We ran several experiments using simulated *Caenorhabditis elegans* reads and a simulated genetic linkage map, real *Arabidopsis thaliana* reads and simulated genetic linkage map, real human reads and a simulated genetic linkage map, and using real reads and a real genetic linkage map for an additional *C. elegans* data set [17, 18] and a nine-spined stickleback *Pungitius pungitius* data set from a recent assembly project [19, 20]. *C. elegans*, *A. thaliana*, and human have good quality reference genomes which allow us to evaluate the correctness of the produced assemblies accurately.

The *P. pungitius* data set allows us to evaluate the assemblers also on a data set where both reads and genetic linkage maps are real but due to the unavailability of a good quality reference genome, the correctness of the assemblies needs to be evaluated with more indirect means such as gene completeness and mappability of reads back to the assembly as explained in more details below. The *C. elegans* data set with real genetic linkage map and reads gives additional weight to any conclusions drawn from the indirect evaluation. The details of the read sets are shown in Table 1.

The long reads for *C. elegans* were simulated with 40x coverage using SimLoRD v1.0.4 [21]. The genetic linkage maps for *C. elegans*, *A. thaliana*, and *H. sapiens* were simulated by randomly positioning markers until 100,000, 50,000, and 700,000 markers respectively had been placed. Of those markers, the ones that are less than 20 bp away from the previous are removed. Bins were assigned to markers by starting from the first marker in the first chromosome and adding markers to the same bin until a marker is at least 200 bp away and then moving to the next bin. The real genetic linkage maps of *C. elegans* assigns a physical position for each marker in the genetic linkage map and thus does not divide the markers into bins. Therefore, we have interpreted the genetic linkage map data as each marker having its own bin.

**Table 1:** Characteristics of the read data sets and genetic maps used in the experiments

Organism	# of reads	Mean read length (bp)	Total read length (Mbp)	Coverage	# of markers	# of bins
*C. elegans* (sim)	478,836	8,214	3,933	40	98,978	81,788
*C. elegans* (real)	3,316,106	8,801	29,185	291	388,202	–
*A. thaliana*	1,135,065	9,475	10,755	90	49,617	45,930
*P. pungitius*	10,918,547	4,948	54,025	115	76,036	34,845
*H. sapiens*	25,986,153	8,916	231,694	76	996,603	936,534

The *C. elegans* (sim) reads were simulated with SimLoRD and the *C. elegans* (sim), *A. thaliana*, and *H. sapiens* genetic linkage maps were simulated by randomly positioning the markers on the genome. The *C. elegans* (real), *A. thaliana*, *P. pungitius*, and *H. sapiens* reads are real PacBio reads

We ran all the methods on a cluster with 16 cores reserved with default settings for all tools. The produced assemblies were evaluated with QUAST v5.0.2 [22] and BUSCO v5.2.2 [23]. QUAST reports genome fraction, misassemblies, and NGA50 values by aligning the assemblies to a reference genome. Additionally QUAST aligns the reads to the assemblies and reports the fraction of reads that can be mapped to the assemblies. For the *P. pungitius* assemblies we do not report misassembly and NGA50 statistics because a high quality reference sequence is not available.

BUSCO reports the number of single-copy orthologs from a gene set it can detect from the assemblies. We used the Nematoda, Brassicales, Actinopterygii, and primates gene sets (odb10 for all sets) for the BUSCO evaluation of *C. elegans*, *A. thaliana*, *P. pungitius*, and *H. sapiens*, respectively. We report the runtime of the methods as the total wall clock time and the memory usage is reported as the peak memory usage during execution.

### The minimum leaf size

The most important parameter in our method is the minimum leaf size, i.e. the minimum number of reads assigned to each leaf in the hierarchical tree. We experimented with different leaf sizes for both the simulated and the real data. The results of these experiments are shown in Tables 2 and 3 . We tried different minimum leaf sizes ranging from 0.1 to 5% of the reads. On the simulated data, the best assemblies regarding the number of contigs and the number of misassemblies are achieved with medium sized leaves. The genome fraction goes down as the leaf size increases. The proportion of mapped reads is similar across all leaf sizes. The runtime is fairly stable and the peak memory usage increases as the leaf size increases because assembling the leaves needs more memory for large leaves as there is more data per leaf to assemble. The NGA50 is less stable. This is likely due to the low number of contigs as in such scenarios a single join of two contigs can have a big effect on the NGA50. The BUSCO completeness is highest for assemblies that also have high NGA50 values but also the assembly produced with the smallest leaf size has a high BUSCO completeness score. For further comparisons with other methods, we will use the assemblies producing the highest NGA50.

On the real *P. pungitius* data the best N50 value is achieved with the minimum leaf size 1.5% of all reads. The medium minimum leaf sizes also achieve the longest assemblies and the most accurate assemblies as measured by BUSCO completeness and the proportion of mapped reads. The number of contigs is slightly smaller for the largest minimum leaf size but this assembly has a lower N50 value and the total length of the assembly is lower than the reference GenBank assembly (GCA_902500615.3). For comparisons with other methods we will use the leaf size 1.5% producing the best N50 value.

For both of our data sets good minimum leaf size is around 1.5% of the reads. However, we note that a denser genetic linkage map allows for smaller leaves. Similarly high coverage of the reads would increase the optimal number of reads per leaf.

**Table 2:** The effect of the minimum leaf size on the assembly of the simulated *C. elegans* data

Min leaf size (% of reads)	# of contigs	NGA50 (bp)	Genome fraction	Misassemblies	BUSCO Complete (%)	Reads mapped (%)	Runtime (min)	Peak memory (MB)
0.1%	42	3,901,186	99.699	11	97.8	99.78	60	1564
0.5%	34	4,282,525	99.564	11	93.4	99.76	55	487
1.0%	30	4,274,710	99.592	9	93.4	99.77	53	901
1.5%	31	5,901,436	99.595	14	97.2	99.78	51	1334
2.0%	37	4,691,641	99.604	15	93.5	99.77	53	1776
2.5%	38	3,900,976	99.568	12	93.3	99.78	54	2226
5.0%	39	5,335,812	99.571	16	98.1	99.78	40	3954

**Table 3:** The effect of the minimum leaf size on the assembly of the real *P. pungitius* data. The length of the scaffold level reference assembly (GCA_902500615.3) is 466 Mbp

Min leaf size (% of reads)	# of contigs	N50 (bp)	Total length (bp)	BUSCO complete (%)	Reads mapped (%)	Runtime (h)	Peak memory (MB)
0.1%	1945	918,119	453,155,823	88.0	92.6	13.34	6,212
0.5%	1084	1,799,563	489,091,741	91.3	93.52	14.3	8,289
1.0%	884	1,877,796	511,024,231	92.1	93.95	13.88	11,680
1.5%	790	2,119,727	503,905,067	92.5	93.91	13.44	16,001
2.0%	779	2,059,129	499,019,519	91.7	93.82	11.98	17,884
2.5%	784	2,027,447	481,429,790	91.7	93.65	17.95	22,713

### Map density

To study the effect of the density of the genetic linkage map, i.e. the number of markers, we simulated maps with different numbers of markers using the *C. elegans* data. We ran both HGGA and Kermit on these data sets. The results are shown in Table 4. HGGA is using closest marker coloring in all cases. We see that once the map is dense enough, the quality of the assembly hardly changes because once this threshold is reached, the reads originating from repeat regions are assigned to different leaves resulting in a good quality assembly which cannot be further improved by more fine grained division of the reads. For Kermit this happens when the density reaches 50k and for HGGA when the density reaches 10k. HGGA is less sensitive to the density because we color the reads using the marker which is closest to the alignment of the read when the alignment does not contain any markers. Kermit, on the other hand, colors these reads by propagating the colors in the overlap graph which can lead to ambiguous colorings.

**Table 4:** The effect of the map density on the assembly of the *C. elegans* data

Method	# of markers	# of contigs	NGA50 (bp)	Genome fraction	Misassemblies	BUSCO Compl. (%)	Reads mapped (%)	Runtime (min)	Peak memory (MB)
Kermit	1k	850	89,141	74.315	13	73.4	91.84	21	11,943
Kermit	10k	733	82,640	68.808	16	67.8	90.69	21	11,809
Kermit	20k	216	818,928	95.417	9	93.8	98.09	22	12,434
Kermit	50k	69	3,450,849	99.539	12	98.0	99.74	23	12,542
Kermit	100k	61	3,476,344	99.563	11	98.3	99.75	23	12,543
Kermit	150k	64	3,450,700	99.555	12	98.1	99.77	23	12,555
Kermit	200k	64	3,476,344	99.563	11	98.3	99.75	23	12,542
Kermit	500k	64	3,476,344	99.563	11	98.2	99.75	23	12,544
HGGA	1k	69	2,488,265	95.627	8	93.8	97.63	38	1902
HGGA	10k	44	3,668,792	99.698	9	97.9	99.75	40	1837
HGGA	20k	44	3,668,641	99.680	10	95.6	98.52	42	1835
HGGA	50k	46	3,668,702	99.708	9	95.9	98.52	42	1827
HGGA	100k	49	3,668,667	99.646	9	97.8	99.78	42	1874
HGGA	150k	51	3,668,731	99.669	8	98.1	99.75	42	1886
HGGA	200k	52	3,869,053	99.568	8	96.2	99.36	43	1833
HGGA	500k	47	3,668,735	99.652	13	98.0	99.76	48	1837

### Assembly height

To evaluate the effects of assembly in the internal nodes, we also ran QUAST and BUSCO on all the contigs generated during the hierarchical assembly process. The results are shown in Table 5. It should be noted that the height of the assembly trees is not an adjustable parameter of the method, rather it is derived from the width of tree, i.e. the number of leaf nodes. The width of the trees is controlled by both the minimum leaf size and the density of the map.

As expected, the contigs get joined to form longer and longer sequences as the assembly process moves up the trees. The leaf contigs contain duplicated sequences by design as the leaves are forced to overlap. Most of this duplication is removed as contigs are joined and contigs that are contained in the joined sequences are removed. This can be seen in the number of contigs plummeting in the first two levels of the internal node assemblies. The number of misassemblies increases after the final assembly, which takes the chromosomally separated trees and attempts to find possible overlaps due to errors in the map.

**Table 5:** The effect of assembly in the internal nodes on the *C. elegans* data

Height	# of contigs	NGA50 (bp)	Genome fraction	Misassemblies	BUSCO Complete (%)	Reads mapped (%)
leaves	221	2,840,136	99.619	17	98.6	99.88
1	112	3,323,225	99.599	17	97.7	99.84
2	71	3,473,215	99.571	13	98.1	99.81
3	59	3,540,478	99.551	12	97.6	99.79
4	51	3,549,527	99.551	12	97.6	99.78
root	31	5,901,436	99.595	14	97.2	99.78

### Comparison to previous work

We compared HGGA to miniasm [1] which uses only the reads and Kermit [11] which uses both the reads and the genetic linkage map. We ran Racon [24] to polish the assemblies produced by miniasm and Kermit since they do not implement a consensus phase. We note that HGGA uses Racon to polish the leaf assemblies and thus produces a polished assembly. We limited the comparison to these tools because all of them use the same module for assembling the reads and thus from this comparison we can see how the integration of the genetic linkage map improves assembly. The results of the comparison on the simulated *C. elegans*, *A. thaliana*, *H. sapiens*, and real *C. elegans* and *P. pungitius* data are shown in Tables 6, 8, 9, 7, and 10 , respectively.

Table 6 shows that on the simulated *C. elegans* data, both Kermit and HGGA are able to improve upon the miniasm assembly which uses only the read data. HGGA produces 63% less contigs than Kermit and twice as large NGA50 value as Kermit but also seven more misassemblies. Kermit also has the highest BUSCO completeness score, but slightly higher number of reads can be mapped back to the HGGA assembly. Kermit is faster but HGGA uses less memory. The results for *C. elegans* assemblies with real genetic linkage maps and reads are shown in Table 7. The results mostly agree with those of the simulated setting, albeit the differences between the tools are less drastic due to being less perfect.

The results on the *A. thaliana* data, where the reads are real but the genetic linkage map is simulated, are shown in Table 8. Kermit produces the smallest number of contigs. However, HGGA produces an assembly with 1.6 times higher NGA50 value, while the NGA50 value of the Kermit assembly is actually slightly smaller than for the miniasm assembly. The assembly produced by Kermit has the smallest number of misassemblies but less than 90% of reads can be mapped back to it, whereas over 95% of the reads map back to the HGGA and miniasm assemblies. The number of misassemblies in the HGGA assembly is still 30% lower as compared to the miniasm assembly and the BUSCO completeness score is highest for HGGA. Similar to the *C. elegans* data Kermit is faster but HGGA uses less memory.

On the *H. sapiens* data shown in Table 9, HGGA produces a much more contiguous assembly compared to the other tools, as shown by the number of contigs and NGA50 value. As with the other datasets, Kermit produces the fewest misassemblies. However, this experiment shows that HGGA scales well to larger data sets as its memory usage remains low (69 GB as compared to more than 560 GB used by miniasm and Kermit) and it is also the fastest method.

Table 10 shows that on the real *P. pungitius* data, HGGA and Kermit both again improve over miniasm. Kermit has the lowest number of contigs but gives shortest assembly overall. HGGA has only slightly less contigs compared to Kermit but the contigs are longer which leads to the highest N50 value. The accuracy of all assemblies are similar with the miniasm assembly having slightly higher BUSCO completeness score than the other assemblies, and HGGA having the highest number of reads mapping to it. The runtime of all the tools is similar while HGGA uses only 10% of the memory used by the other tools.

HGGA produces an assembly which is longer than the reference genome, whereas the miniasm assembly is roughly of the same size and the Kermit assembly is smaller. To get a further estimate of the genome size, we computed the number of distinct 51-mers with abundancy above five in Illumina reads produced for this same genome, which yielded a genome size estimate of 450 million. The assemblies produced by miniasm, Kermit, and HGGA have 429 million, 413 million, and 431 million distinct 51-mers, respectively. As expected, these numbers are lower since the Illumina reads contain 51-mers from both haplotypes, whereas the assemblers attempt to produce a single haplotype. To further analyse the *k*-mer spectrum of the assemblies and reads, we generated the copy number spectrum plots for the three assemblies which are shown in the supplementary material (Additional file 1: Figure S1). This analysis shows that HGGA has more duplicated *k*-mers that the other assemblies and thus the longer length is due to duplicated sequence.

**Table 6:** Comparison of HGGA, miniasm, and Kermit on the simulated *C. elegans* data

Method	# of contigs	NGA50 (bp)	Genome fraction	Misassemblies	BUSCO complete (%)	Reads mapped (%)	Runtime (min)	Peak memory (MB)
Miniasm	126	1,982,361	99.443	10	98.1	99.75	20	18,332
Kermit	83	2,819,353	99.535	7	98.3	99.75	23	19,578
HGGA	31	5,901,436	99.595	14	97.2	99.78	51	1,334

**Table 7:** Comparison of HGGA, miniasm, and Kermit on the *C. elegans* data with real genetic linkage map and reads

Method	# of contigs	NGA50 (bp)	Genome fraction	Misassemblies	BUSCO complete (%)	Reads mapped (%)	Runtime (h)	Peak memory (MB)
Miniasm	472	1,582,439	99.478	420	95.2	94.43	5.52	88,371
Kermit	95	1,864,384	99.187	197	95.8	93.41	4.88	88,028
HGGA	217	1,927,968	99.072	195	95.1	94.61	9.07	9,101

**Table 8:** Comparison of HGGA, miniasm, and Kermit on the *A. thaliana* data with real reads and simulated genetic linkage map

Method	# of contigs	NGA50 (bp)	Genome fraction	Misassemblies	BUSCO Complete (%)	Reads mapped (%)	Runtime (h)	Peak memory (MB)
Miniasm	712	2,552,623	98.766	346	84.5	96.63	2.37	34,128
Kermit	123	2,552,489	98.185	174	85.1	89.07	2.08	34,486
HGGA	136	4,173,314	98.247	242	86.3	95.87	3.41	10,050

**Table 9:** Comparison of HGGA, miniasm, and Kermit on the *H. sapiens* data with real reads and simulated genetic linkage map

Method	# of contigs	NGA50 (bp)	Genome fraction	Misassemblies	BUSCO complete (%)	Reads mapped (%)	Runtime (h)	Peak memory (MB)
Miniasm	8,789	692,902	89.761	3,669	76.5	61.37	237.84	565,309
Kermit	4,503	1,050,164	90.069	762	77.9	60.65	239.29	565,307
HGGA	2,204	6,814,538	93.181	3,004	86.5	70.45	37.46	69,492

**Table 10:** Comparison of HGGA, miniasm, and Kermit on the real *P. pungitius* data. The length of the scaffold level reference assembly (GCA_902500615.3) is 466 Mbp

Method	# of contigs	N50 (bp)	Total length (bp)	BUSCO complete (%)	Reads mapped (%)	Runtime (h)	Peak memory (MB)
Miniasm	1,873	1,182,753	461,795,357	92.7	93.58	13.49	165,716
Kermit	833	1,392,886	432,823,234	92.1	93.08	13.19	165,061
HGGA	790	2,119,727	503,905,067	92.5	93.91	13.44	16,001

## Discussion

We have presented HGGA, a method for assembling read data with the help of genetic linkage maps. Our experiments show that the number of contigs decreases 12-80% as compared to an assembly using only read data. When compared to Kermit, our previous method for assembling read data with genetic linkage maps, the number of contigs increases on the *A. thaliana* and real*C. elegans* data sets but decreases on the other three data sets. HGGA produces up to 9.8 times longer NGA50 values as compared to a read only assembly with miniasm and up to 6.5 times longer NGA50 when compared to Kermit.

On the simulated *C. elegans* data all methods produce few misassemblies and on the *A. thaliana* and human data HGGA produces more misassemblies than Kermit but less than miniasm, whereas on the real *C. elegans* data set HGGA produces a similar number of misassemblies as Kermit but less than miniasm. The runtime of HGGA is longer than the runtime of previous methods on the simulated and real *C. elegans* and *A. thaliana* data sets but similar on the *P. pungitius* data and less than a sixth on *H. sapiens* data. On all data sets, HGGA uses significantly less memory as the reads are assembled one subset at a time and thus do not reside in the memory simultaneously.

We assume here that the genetic linkage map has been constructed for a draft assembly. Recently, a tool called AFLAP [25] has been published which builds a genetic linkage map in a reference-free manner using *k*-mer data. By integrating AFLAP output with HGGA  we could avoid the need for a draft assembly for the genetic linkage map construction. However, this would require localizing the markers on the highly erroneous PacBio reads based on the *k*-mers output by AFLAP instead of our current practise of aligning the reads to the draft assembly.

In this work, we only consider contig assembly, i.e. assembling the reads into contiguous sequences without gaps. Our method does not do scaffolding, which is the process of ordering the contigs into scaffolds where contigs are separated by gaps. Thus we did not compare HGGA against scaffolding methods which use genetic linkage maps for scaffolding. Such tools include for example Chromonomer [5] and Lep-Anchor [6]. Because these tools only scaffold the contigs, the contigs themselves do not change and thus the contig statistics remain the same as for the input set of contigs. Furthermore, such a scaffolding method could be run after HGGA to further increase the contiguity of the assembly.

Our current implementation only supports genetic linkage maps. As further work, it would be interesting to extend the implementation to use optical maps or Hi-C data. This would only require developing a method for dividing the reads into leaves based on the different kind of guide data. The hierarchical assembly of the leaves and the internal nodes of the hierarchical tree would remain the same.

## Conclusions

We have presented a framework for integrating additional data such as genetic linkage maps, optical maps, and Hi-C data to genome assembly, and implemented it for genetic linkage maps. The key insight of our method is to use the additional data to partition the reads into overlapping subsets and assemble the subsets independently. Because the assembly of the subsets is independent, our approach is inherently easy to parallelize beyond a single machine. Our implementation of the approach for genetic linkage maps shows that it improves the contiguity of the assembly on both simulated and real data.

## Methods

### Genomic distance function

Here, we show how to apply the idea of assembly guiding to any data that can be represented with a measure of positional similarity between reads. First, we define a distance function between two reads that gives the 1-dimensional genomic distance in base pairs. Using hierarchical clustering methods, we can then construct a hierarchy tree using this distance function.

Given two reads $R_1$ and $R_2$ that originate from positions $p_1$ and $p_2$ in the genome, the genomic distance $D(R_1,R_2) = |p_1-p_2|$. In practice, we do not have access to the exact distance function *D* but we attempt to estimate it with the guide data.

For reference guided assembly, we can construct a genomic distance function for the reads by aligning the reads to the reference and computing distances between alignments. Assuming a high quality reference and good alignments for the reads, this gives a good estimate of the distance measure. The drawback is the requirement for high quality reference genome.

Genetic linkage maps are constructed relative to some draft assembly and so we have access to the draft assembly and the markers of the map are positioned on the draft assembly. Thus to estimate the genomic distance function using genetic linkage maps, we align reads to the map-relative draft assembly of the genome and find all overlapping markers in the map. We then apply all overlapping markers to reads and compute a distance based on the lists of markers. While the markers will not give a basepair level of accuracy for distance, they do give a good relative distance.

Optical maps are constructed by applying a restriction enzyme on a DNA molecule. The restriction enzyme cuts or nicks the DNA at a specific DNA pattern called restriction site. The fragment lengths between the restriction sites are then measured and they form the optical map. An optical map of a genome thus is a sequence of fragment lengths. In principle, reads could be *in silico* digested to a sequence of fragment lengths and then mapped to the optical map. However, the reads are too short and the optical maps too sparse for this to work in practice [12]. Thus to localize reads on an optical map, they need to be assembled first into draft contigs, which then can be mapped to the optical map. Since alignments of the reads to the contigs are known, the mapping of reads to the optical maps can be found via the contig mappings [12]. The genomic distance between two reads can then be estimated based on their mappings to the optical map.

Once we have an estimate of the genetic distance function for the reads, we can use any hierarchical clustering method to produce a dendrogram for the reads. We can then cut the dendrogram at a suitable depth to produce the hierarchical tree for assembly. The memory and time required for naively constructing both the distance function and hierarchy are both quadratic over the number of reads. As such, a different approach is required in practice.

### Hierarchical tree

For genetic linkage maps, we can exploit the linear ordering of the bins to fill leaf nodes with a linear scan of the reads. We align all reads to the map-relative assembly and find all overlapping markers in the map for each read. Each of the markers belongs to a bin of the genetic linkage map, and so the bins of the markers overlapping the alignment of a read are associated with that read. Alternatively, if the genetic linkage map is sparse and all read alignments do not overlap any markers, we can find the closest marker for the alignment of each read and define the bins associated with a read as the bins of the set of markers that are roughly the same distance away from the alignment of the read as the closest marker.

We then sort the reads based on their associated bins and make a linear scan through the sorted read set. We add entire bins to the current preliminary leaf node until a minimum number of reads is reached and then move on to fill the next preliminary leaf. The sorting can be done by radix sorting and thus the whole process of constructing the hierarchical tree in this way only takes linear time and is far more practical than constructing a genomic distance function and building the hierarchical tree with hierarchical clustering.

**Fig. 2:**
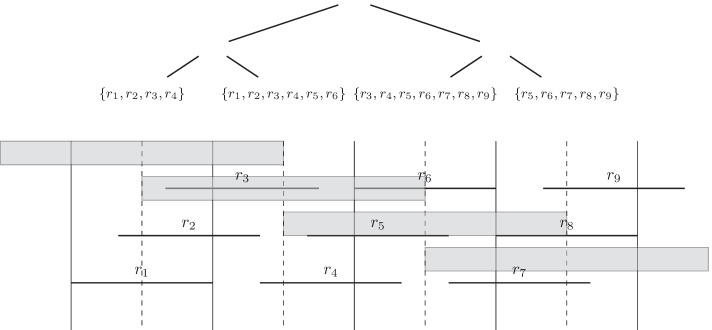
An example of how reads, shown as black horizontal lines, are assigned to leaf nodes. Reads have each been assigned to one or more preliminary leaf nodes (shown in black vertical lines). Each preliminary leaf gets further split in half (shown as dashed vertical lines). These halves are then merge back together with their neighbors (shown as grey rectangles) and assigned to the final leaf nodes in their order of appearance

In order to guarantee that leaf assemblies have sufficient overlap with each other, we additionally overlap all preliminary leaf nodes with their neighbors. We split each preliminary leaf node in half into two blocks and take the union of four consecutive blocks as a final overlapped leaf node. In other words, each leaf node now is the union of a preliminary leaf node, the left half of the right preliminary leaf, and the right half the left preliminary leaf. Figure 2 illustrates the process. This also has the added benefit of making the coverage in the leaves higher and more even.

### Assembly

The first step in our assembly flow is assembling each leaf node in the hierarchy independently. Any assembly pipeline that is suitable for the read data is valid here. We use miniasm [1] for assembly and Racon [24] for polishing the leaf assemblies given the long read data we use.

After the leaf assemblies, we start going up the hierarchy. For each unassembled node in the tree, we take its (up to) two children and find all overlaps between the contigs produced in the child nodes, build an overlap graph, and find an assembly path. This process looks deceptively similar to a regular assembly flow that we use in the leaves. However, not only are the input sequences very long and error corrected, we also expect there to be a very small number of input sequences. As most modern assembly pipelines have to be optimized for as large number of input sequences as possible, they are required to be more conservative in terms of time per sequence.

For our assembly flow in internal nodes, we use minimap2 [26] to find all pair-wise overlaps and filter out self-loops, short overlaps (< 10 kbp), and contained overlaps. We then build our overlap graph from the remaining overlaps. To correctly handle the double strandedness of the genome, we use the following undirected graph to simulate a bidirected overlap graph. Each contig *u* in the graph is represented by two vertices, $u_s$ and $u_e$, which represent the start of the contig and the end of the contig, respectively, and a contig edge $(u_s,u_e)$. For each overlap between two contigs, *u* and *v*, we add an overlap edge as follows:

- If the suffix of *u* overlaps with the prefix of *v*, we add the edge $(u_e,v_s)$.
- If the suffix of *u* overlaps with the prefix of the reverse complement of *v*, we add the edge $(u_e,v_e)$.
- If the suffix of the reverse complement of *u* overlaps with the prefix of *v*, we add the edge $(u_s,v_s)$.
- If the suffix of the reverse complement of *u* overlaps with the prefix of the reverse complement of *v*, we add the edge $(u_s,v_e)$.

**Fig. 3:**
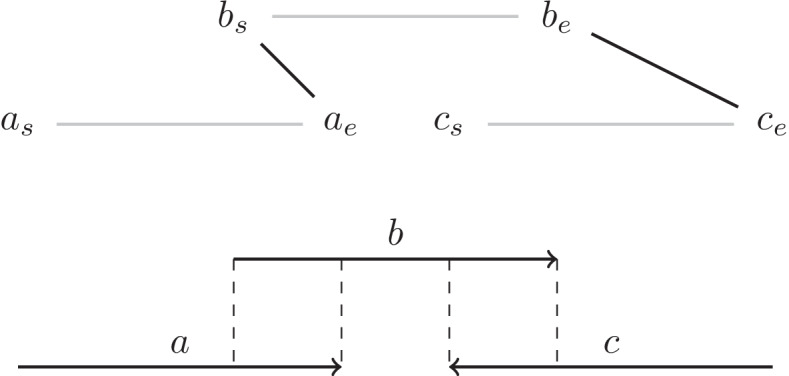
A bidirected overlap graph corresponding to overlaps between contigs *a*, *b*, and *c*. The contig edges are shown in gray and the overlap edges in black. An assembly path through the graph alternates betweem contig edges and overlap edges. In this graph the path ${a_s,a_e,b_s,b_e,c_e,c_s}$ is an assembly path

An example of a bidirected overlap graph is shown in Fig. 3.

Longer super-contigs can now be read from this graph as paths where contig and overlap edges alternate. If we traverse a contig edge in the direction $(u_s\rightarrow u_e)$, the contig is added to the supercontig in forward orientation, and if we traverse a contig edge in the direction $(u_e\rightarrow u_s)$, the contig is added to the super-contig in reverse complementary orientation. We start from the node in the graph with the lowest number of edges and first traverse the contig edge. Then we choose the edge from the node corresponding to the largest overlap between the contigs and continue traversing contig and overlap edges alternatingly until there is no overlap edge to follow. We repeat this until all maximal super-contigs are found.

As the reads get separated into their own trees, no overlaps are observed on pairs of reads that get mistakenly identified as being in different chromosome in the final assembly. To attempt to combat this issue, we perform one final assembly step using the super-contigs from the root nodes of each tree in the hierarchy. This assembly step can, and by default does, use stronger parameters such as requiring longer overlaps and longer sequences.

### Evaluation of assemblies

We use QUAST [22] and BUSCO [23] to evaluate the correctness of the produced assemblies. Here we explain the metrics used in the experiments in detail.

QUAST computes several metrics based solely on the set of contigs. When given a reference genome, it aligns the contigs against the reference genome using Minimap2 [26] and evaluates the correctness of the assembly based on the alignments. Additionally, a set of reads can be provided to QUAST and then it maps the reads to the assembly using BWA [27] and reports statistics based on the read mappings. In particular, we report the following statistics for data sets with a reference genome:

- **# of contigs:** The number of contigs in the assembly.
- **NGA50:** NG50 is the shortest contig length such that half of the genome is covered by contigs of length at least the NG50 size. When computing NGA50, the contigs are first aligned to the reference genome and then broken at each position where a misassembly occurs. NGA50 is then the NG50 value of this broken set of contigs.
- **Genome fraction:** The percentage of bases in the reference genome that are covered by at least one alignment of a contig to the reference genome. Contigs from repetitive regions are allowed to align to several positions in the reference genome.
- **Misassemblies:** The number of positions in the contigs such that the sequence to the left of the positions and the sequence to the right of the position align 1 kbp away from each other or the two alignments of the two sequences overlap by at least 1 kbp or they align to different strands or chromosomes.
- **Reads mapped:** The percentage of reads mapping to the assembly.

On the *P. pungitius* data set, where a reference genome is not available, we cannot compute NGA50, genome fraction, and the number of misassemblies. In addition to the number of contigs and reads mapped, we then report

- **N50:** The shortest contig length such that half of the assembly is covered by contigs of length at least the N50 size.
- **Total length:** The total length of the contigs in the assembly.

We use BUSCO [23] to further evaluate the completeness of assemblies. This is especially important for the *P. pungitius* data set which lacks a good quality reference genome and thus genome fraction cannot be computed for the *P. pungitius* assemblies. However, we provide the BUSCO completeness score for assemblies on all data sets to facilitate comparisons across the data sets. BUSCO evaluation is based on universal single-copy orthologs which are genes expected to be present across different species. For further accuracy, BUSCO comes with tailored gene sets for different clades. Thus we used the Nematoda, Brassicales, Actinopterygii, and primates gene sets for the BUSCO evaluation of *C. elegans*, *A. thaliana*, *P. pungitius*, and *H. sapiens*, respectively. For each assembly, we report the BUSCO completeness score which is the percentage of the universal single copy orthologs that were found in the assembly in one or more copies.

## Supplementary information

**Additional file 1.**Figure S1: The *k*-mer spectrum of the *P. pungitius* Illumina reads (a) and the copy number spectrum plots of the *P. pungitius* assemblies produced by miniasm, Kermit, and HGGA. The copy number spectrum plots divide the *k*-mersinto subsets according to their copy number in the assembly. For each subset, the spectrum is then plotted according to the abundancies of the *k*-mers in the read set.

## Data Availability

HGGA is freely available at https://github.com/rikuu/hgga. The *C. elegans* and *A. thaliana* reference genomes were downloaded from NCBI (https://www.ncbi.nlm.nih.gov/genome/41?genome_assembly_id=43998, accession codes NC_003279.8, NC_003280.10, NC_003281.10, NC_003282.8, NC_003283.11, NC_003284.9 for *C. elegans* and https://www.ncbi.nlm.nih.gov/genome/4?genome_assembly_id=454618, NC_003070.9, NC_003071.7, NC_003074.8, NC_003075.7, NC_003076.8 for *A. thaliana*). The *P. pungitius* draft genome was downloaded from NCBI (https://www.ncbi.nlm.nih.gov/assembly/GCA_902500615.3, accession code GCA_902500615.3). The human T2T reference genome was downloaded from NCBI (https://www.ncbi.nlm.nih.gov/assembly/GCA_009914755.3, GCA_009914755.3). The real *C. elegans* reads were downloaded from SRA (accession codes SRX4459462, SRX4459460, and SRX4459459) and the real *C. elegans* genetic linkage map is from [18]. The *A. thaliana* reads are available at https://downloads.pacbcloud.com/public/SequelData/ArabidopsisDemoData. The *P. pungitius* reads were downloaded from ERA (accession code ERR3569182) and the genetic linkage map is from [20]. The Illumina reads of *P. pungitius* were also downloaded from ERA (accession codes ERR3618123 and ERR3618124). The *H. sapiens* reads were downloaded from SRA (accession codes SRX825577 and SRX825578). The script for generating simulated genetic linkage maps is included in Kermit at https://github.com/rikuu/kermit.

## Abbreviations

HGGA: Hierarchical Guided Genome Assembler
SNV: Single nucleotide variation
GB: Gigabytes
MB: Megabytes
bp: Base pairs
