# Supplementary material for: HGGA: hierarchical guided genome assembler
Source: BMC Bioinformatics. 2022 May 7;23:167. doi: 10.1186/s12859-022-04701-2 (PMC9077837; doi:10.1186/s12859-022-04701-2)
Supplement: Supplementary file 1 — Additional file 1.Figure S1: The k-mer spectrum of the P. pungitius Illumina reads (a) and the copy number spectrum plots of the P. pungitius assemblies produced by miniasm, Kermit, and HGGA. The copy number spectrum plots divide the k-mersinto subsets according to their copy number in the assembly. For each subset, the spectrum is then plotted according to the abundancies of the k-mers in the read set. [file 12859_2022_4701_MOESM1_ESM.pdf]

Supplementary material: HGGA: Hierarchical Guided Genome Assembler

Riku Walve and Leena Salmela

Department of Computer Science, Helsinki Institute for Information Technology HIIT, University of Helsinki

1 Analysis of the k-mer spectrum of *P. pungitius* assemblies

To validate the *P. pungitius* assemblies, we performed k-mer spectrum analysis of the assemblies and Illumina reads (accession codes ERR3618123 and ERR3618124) produced for the same genome. First, we used DSK [2] to compute the k-mer spectrum of the reads using $k = 51$. We then ran Genoscope [3] to estimate the heterozygosity rate of the genome yielding the range 0.201%-0.203% which is in line with the heterozygosity rate of the genome reported by Fang et al. [1]. Finally we used the "-histo2D" option of DSK to generate copy number spectrum plots for the assemblies produced by miniasm, Kermit, and HGGA. The k-mer spectrum of the reads and the copy number spectrum plots of the assemblies are shown in Figure S1.

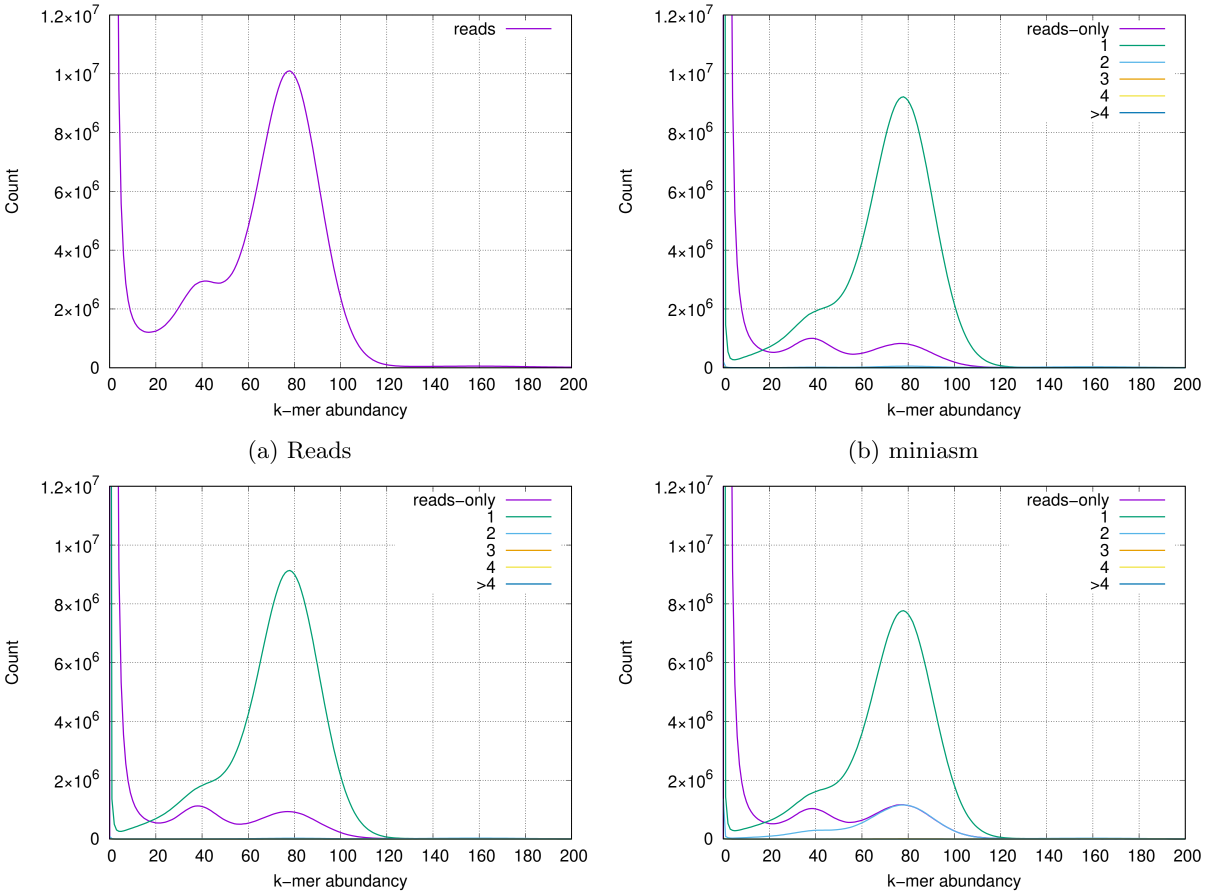

(a) Reads (b) miniasm

(c) Kermit (d) HGGA

Figure S1: The k-mer spectrum of the *P. pungitius* Illumina reads (a) and the copy number spectrum plots of the *P. pungitius* assemblies produced by miniasm, Kermit, and HGGA. The copy number spectrum plots divide the k-mers into subsets according to their copy number in the assembly. For each subset, the spectrum is then plotted according to the abundancies of the k-mers in the read set.

References

[1] Bohao Fang, Petri Kemppainen, Paolo Momigliano, and Juha Merilä. Population Structure Limits Parallel Evolution in Sticklebacks. *Molecular Biology and Evolution*, 38(10):4205–4221, 05 2021. URL: https://doi.org/10.1093/molbev/msab144, arXiv:https://academic.oup.com/mbe/article-pdf/38/10/4205/40449636/msab144.pdf, doi:10.1093/molbev/msab144.

[2] Guillaume Rizk, Dominique Lavenier, and Rayan Chikhi. DSK: k-mer counting with very low memory usage. *Bioinformatics*, 29(5):652–653, 2013.

[3] Gregory W Vurture, Fritz J Sedlazeck, Maria Nattestad, Charles J Underwood, Han Fang, James Gurtowski, and Michael C Schatz. GenomeScope: fast reference-free genome profiling from short reads. *Bioinformatics*, 33(14):2202–2204, 03 2017. URL: https://doi.org/10.1093/bioinformatics/btx153, arXiv:https://academic.oup.com/bioinformatics/article-pdf/33/14/2202/25157257/btx153.pdf, doi:10.1093/bioinformatics/btx153.
